# High-intensity focused ultrasound: an innovative approach for micro-manipulation of demineralized dentine

**DOI:** 10.1186/s12967-024-06008-7

**Published:** 2025-01-08

**Authors:** Sheetal Maria Rajan, Barsha Shrestha, Amr Fawzy

**Affiliations:** Dental School, The University of Western Australia, 17 Monash Avenue, Nedlands, WA 6009 Australia

**Keywords:** High-intensity focused ultrasound (HIFU), Dentine, Carious dentine, Minimally invasive dentistry, Cariogenic biofilm

## Abstract

**Background:**

Treatment of deep carious lesions poses significant challenges in dentistry, as complete lesion removal risks compromising pulp vitality, while selective removal often reduces the longevity of restorations. Herein, we propose a minimally invasive approach using High-Intensity Focused Ultrasound (HIFU) for microscale removal of carious dentine. Concurrently, HIFU’s antimicrobial effects against associated cariogenic biofilms and the corresponding thermal and biological impacts on surrounding tissues were investigated.

**Methods:**

A total of 238 sound human molars were utilized, with 203 samples of artificial carious-simulated dentine (ACSD) prepared for HIFU exposure. HIFU (250 kHz) was applied at 20 W for varying durations (60, 120, and 180 s). The acoustic waves were administered via a collimated cone coupled to the dentine surface using ultrasonic gel.

**Results:**

Advanced characterization techniques including scanning electron microscopy (*n* = 5/group), Raman spectroscopy, atomic force microscopy, and nano-indentation (*n* = 5/group), demonstrated HIFU’s effectiveness in removing demineralized collagen-fibrils. This was reflected in the increased mineral content, nano-hardness, and reduced elastic-modulus of ACSD lesions. Micro-CT (*n* = 6/group) confirmed the increase in mineral density post-HIFU exposure. Confocal microscopy of Rhodamine-B stained ACSD (*n* = 5/group) quantified the depth of dentine microscale removal post-HIFU exposure in a time-dependent manner. HIFU’s potent anti-biofilm effect (*n* = 9/group) against *Streptococcus mutans* biofilms was evidenced by microscopic characterizations and significant reductions in metabolic-activity and colony-forming units. Furthermore, HIFU promoted the proliferation of dental pulp stem cells (*n* = 3/group) while maintaining the associated temperature-rise within the physiological tolerance.

**Conclusion:**

HIFU’s potential as an innovative, minimally invasive, non-ionizing tool for dentine carious lesion micromanipulation was demonstrated through the interaction between focused acoustic waves and dentine, warranting further studies for future clinical translation in restorative and/or preventive dentistry.

## Introduction

Dental caries, commonly known as tooth decay, is a prevalent oral health issue caused by an imbalance in the oral microbiome, characterized by acidogenic and aciduric bacteria thriving in the presence of frequent carbohydrate consumption [1–3]. The metabolic by-products of these bacteria lead to enamel demineralization, initiating a cascade of events that can progress to dentine invasion, further demineralization, and eventual cavitation if left untreated [1]. Deep caries, pose a risk of pulp exposure, complicating treatment decisions due to challenges in accurately assessing caries depth and residual dentine thickness [1, 4, 5].

One of the primary challenges in treating dental caries lies in effectively and conservatively removing carious dentine whilst preserving sound and healthy tooth structure. Traditional approaches often involved the complete removal of carious dentine (both infected and affected), to provide a sound mineralized structural base for the restoration and to further arrest the cariogenic activity within the lesion [1, 4, 6]. However, this complete removal approach led to the unnecessary removal of surrounding viable tissues, larger restorations, and compromised tooth integrity [7, 8]. To address these challenges, minimally invasive selective caries removal methods have been developed. These methods aim to remove only the infected and denatured dentine, leaving behind caries-affected dentine to reduce the risk of pulp exposure [6–8]. Removal of carious dentine using selective techniques includes the use of steel/tungsten carbide burs [6, 9], air-abrasion [6, 7, 10], erbium-doped: yttrium aluminium garnet (Er: YAG) lasers [10, 11], photo-activated disinfection (PAD) using tolonium chloride [12, 13], enzymes, and dentine solubilizing agents such as Carisolv™ [10, 14–16]. Moreover, mechanical rotary instruments, while effective, are indiscriminate, and non-selective and can remove more dentine than necessary, particularly in deep lesions [9, 10, 16]. In addition, these instruments are associated with an increased amount of vibrations, heat, and unpleasant noise, often making the patient uncomfortable [16, 17]. Lasers on the other hand are more expensive, need trained personnel [18], have safety concerns due to electromagnetic radiation [19], and are sensitive to brittle surfaces which is not always the case with demineralized dentine collagen [20]. In addition, studies have reported damage to the pulp and deeper cell layers due to wavelength penetration [21, 22], reduction in dentine bonding strength, and hardness [23–25] upon laser irradiation. Whereas, studies using Carisolv™ have revealed a significant decrease in the Vickers hardness value of the retained dentine [15], even though it resulted in no obvious smear layer and open dentinal tubules [14, 26]. The ability to distinguish and remove the infected dentine, often described as the light yellow/ beige coloured wet/moist surface texture, is highly dependent on the operator’s experience [1, 6, 17]. Therefore, having a tool able to manipulate and selectively remove carious dentine at the microscale level, in particular, the soft infected dentine whilst having anti-biofilm properties is of potential clinical significance.

High-Intensity Focused Ultrasound (HIFU) has gained significant interest and demonstrated potential in various fields of medicine, including dentistry. HIFU utilizes high-amplitude ultrasound energy focused precisely on the targeted area, inducing mechanical stress and micro-cavitation within tissues [27–29]. The microbubbles created can erode or ablate tissues due to the liquefying effect with the generation of hydrodynamic pressure gradients, microjets, and micro-streaming [27, 30]. Studies have demonstrated the potential of HIFU in the delivery of drugs through dentinal tubules [31], smear layer removal, dentinal tubule exposure [27], collagen contraction and remodelling [28], and the creation of textured dentine/ smooth root surfaces upon prolonged exposure [32]. In addition, HIFU demonstrates an enhanced bactericidal effect affecting bacterial cell integrity, with minimal surface damage [33–36]. However, the energy penetration and efficacy of these effects are highly dependent on the targeted tissue and operational parameters of HIFU including power, intensity, and duty cycle [27]. Therefore, optimization of HIFU parameters can render a potential tool to selectively remove carious dentine at a microscale level in a minimally invasive manner.

**Fig. 1:**
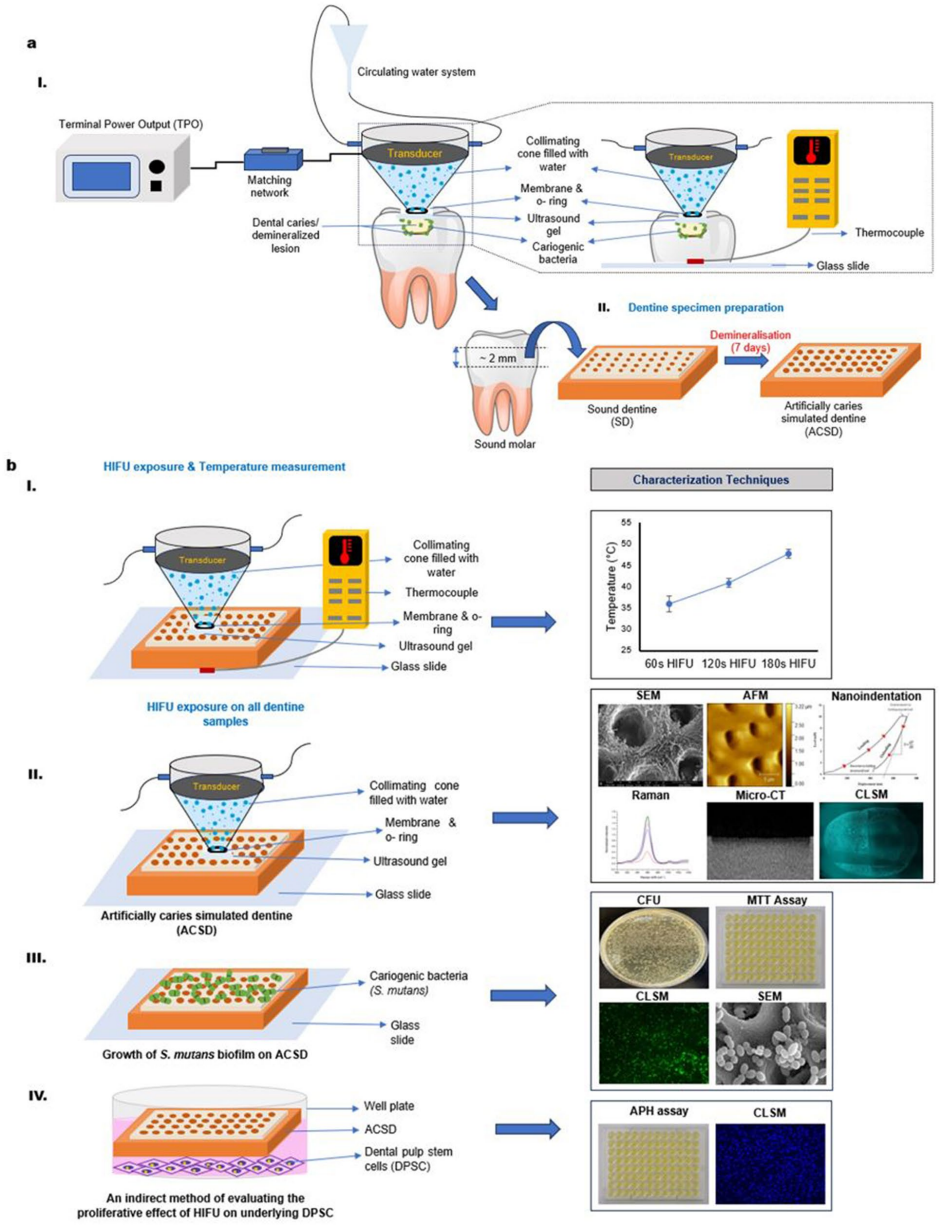
(**aI**) Schematic diagram presenting the proposed concept and the associated experimental set-up of using High-intensity focused ultrasound (HIFU) for microscale manipulation of artificial caries simulated dentine (ACSD) substrate and removal of the associated cariogenic biofilms. The HIFU set-up consists of a terminal power output (TPO), a matching network (50Ω), and a focused ultrasound transducer (250 kHz frequency). The transducer is equipped with a collimating cone filled with degassed water using syringe connections attached to a circulating water system, and the opening is covered with a polyurethane membrane, fixed around the edges of the transducer using an O-ring to secure the seal. A thermocouple placed at the bottom of the dentine specimen was used to measure the temperature change occurring during HIFU exposure. (**aII**) Dentine specimen sectioning and ACSD preparation. (**bI-IV**) schematically summarizing the various characterization methods of the HIFU-exposed dentine specimens

The aim is to investigate the potential of employing HIFU as a minimally invasive tool for microscale manipulation and removal of artificial carious-simulated dentine substrates (ACSD). In addition, to investigate the anti-biofilm effect of HIFU against cariogenic biofilm attached to dentine substrate. Finally, to evaluate the associated temperature rise within the pulp space and the effect on dental pulp stem cell (DPSC) proliferation following HIFU exposure (Fig. 1).

## Materials and methods

Sound human molars (*n = 238*) extracted for dental reasons were used in this study following the patient’s consent and ethical approval from the University of Western Australia Human Research Ethics Committee (grant# RE 2019/RA/4/20/5863). The included molars were cleaned to remove any calculus, soft tissue, and debris, followed by a 10 min rinse with distilled water in an ultrasonic bath. Subsequently, the teeth were disinfected in a 0.5% chloramine-T solution for 24 h, stored in a 0.1% thymol solution, and refrigerated at 4 °C until use. Dentine disc-shaped specimens (~ 2 mm) were sectioned from the mid-coronal dentine using a diamond blade under water coolant (IsoMet, Buehler, Düsseldorf, Germany). The exposed coronal dentine specimens were wet polished with increasing grit sizes from 600 to 4000 SiC papers (Carbimet; Buehler, USA) and cleaned ultrasonically in distilled water for 10 min.

### Artificial caries simulated dentine (ACSD) model

To simulate the artificial caries-simulated lesion, the dentine discs (*n = 203*) were covered with acid-resistant nail varnish on the proximal and bottom surfaces and immersed in 10 mL of demineralizing solution (1.5 mM CaCl_2_, 0.9 mM KH_2_PO_4_, 50 mM acetic acid, 0.02% NaN_3_ with pH value adjusted to 4.5 using NaOH) at 37 °C for 7 days (Fig. 1a) [37]. Following this, the specimens were removed from the container and rinsed thoroughly with distilled water. ACSD lesion formation was verified using micro-computed tomography (micro-CT).

### The experimental setup and HIFU exposure

The proposed concept of using HIFU for microscale manipulation of dentine is schematically presented in Fig. 1a. The HIFU setup consists of a bowl-shaped 64 mm piezo ceramic focused transducer with a resonance frequency of 250 kHz (H-115, Sonic Concepts Inc, WA, USA) equipped with a transparent polycarbonate collimating cone, with a height of 50 mm and radius of 3.1 mm. The transducer has a geometrical focus of 63.2 mm, and a focal depth (measured from the exit plane of the transducer housing rim to geometric focus) of 51.74 mm, respectively. A custom-made electrical matching network was connected between the Terminal Power Output (TPO) (Sonic Concepts Inc, WA, USA) and the transducer. The collimating cone was filled with degassed water using syringe connections, and the opening was covered with a polyurethane membrane, fixed around the edges of the transducer using an O-ring to secure the seal. The geometry of the cone enabled the centre of the focal spot to be on the membrane. A multichannel thermocouple was placed at the bottom of the dentine specimen to measure the temperature change occurring during the HIFU exposure. Each prepared dentine specimens were placed on a glass slide positioned at the focal point of the transducer with 200 µL of sterile ultrasound coupling gel applied on the surface, before being subjected to HIFU exposure. Based on our preliminary investigations, the HIFU parameters were optimized to 20 W power output in continuous mode for varying time points of 60s, 120s, and 180s, to achieve an effective balance between efficacy and clinical feasibility. This setting also ensured that the temperature rise remained within the physiological tolerance of the dental pulp, thereby minimizing any adverse effects on dental pulp stem cells. The associated temperature changes were measured and plotted for each exposure time point (Fig. 1b).

To minimize the impact of dentine variations, the specimens were randomly assigned to the following experimental groups: sound dentine (SD), artificial caries simulated dentine (ACSD), ACSD/HIFU 60s, ACSD/HIFU 120s, and ACSD/HIFU 180s.

### Characterization of dentine specimens

#### Scanning Electron Microscopy (SEM)

The surface morphology of the dentine specimens (*n = 5* per group) was viewed using SEM. The dentine specimens were fixed for 24 h using 2.5% glutaraldehyde + 2% paraformaldehyde in phosphate-buffered saline (PBS) and washed twice with PBS. They were subsequently subjected to a dehydration process involving a series of ascending ethanol concentrations followed by hexamethyldisilazane (HMDS) in a PELCO BioWave microwave operating at 250 W (Pelco BioWave, Ted Pella, Inc., USA). The dried specimens were affixed onto aluminium stubs using copper tape and platinum sputter coated (~ 3 mm) (Polaron SC7640, Quorum Technologies Ltd, U.K). Representative SEM images were captured using Verios (XHR SEM, ThermoFisher Scientific, USA) operated at an accelerating voltage of 5 kV at various magnifications. Additionally, the specimen was fractured, mounted on copper tape, and coated with platinum for viewing the longitudinal sectional view under SEM.

#### Raman spectral analysis

To evaluate the surface chemical composition of the treated dentine specimens (*n = 5* per group) a Raman spectrometer (WITec Alpha300+, GmbH, Ulm, Germany) equipped with a 785-nm diode laser, 20 × 0.5 magnification was employed. Raman signals were acquired using a 600-lines/mm grating centered between 300 and 1800 cm^− 1^. Before data collection, the instrument was calibrated using a silicon disc. From each specimen, 10 different spectra were acquired, each with an integration time of 1 s, and 40 accumulations. Project 5 software (Version 5.1, WITec) was used to analyze the spectral information, smoothing, and manual multiple-point baseline correction was applied to the acquired spectra. The fitting of the Raman spectra was done using the Lorrentz function (Project 5 software, Version 5.1, WITec), and the differences in the degree of the crystallinity, i.e., the full-width at half maximum (FWHM) of phosphate peak at 960 cm^− 1^ were measured [38, 39].

#### Nano-indentation and atomic force microscopy (AFM) imaging

The mechanical properties of the dentine specimens (*n = 5* per group) at the nano-level were characterized using A DualScope™ Scanning Probe & Optical Microscope (DME, Denmark), equipped with a Berkovich diamond indenter (tip radius ~ 20 nm). An array of 5 indents/lines separated by a 10 μm interval in between on a fully hydrated dentine specimen at five different locations was employed. The dentine specimens were introduced to a load force of 0.5 mN, holding time of 10 s between 20 increments and 20 decrements. Subsequently, the nano-hardness and reduced-elastic modulus were calculated using the load function profile average at a compliance of 0.000360 μm/mN.

An Atomic force microscope (Cypher VRS, Oxford Instruments, UK) was used to characterize the surface microtopography of the dentine specimens (*n = 5* per group). The AFM was operated at high resolution using contact mode. A total of five different areas across the dentine specimen were measured using the following parameters: resolution of 256 lines, scan area of 20 μm x 20 μm, scan rate of 4.34 Hz, setpoint of 1.517 V, and an integral gain of 49.23.

As the above three tests are non-destructive, the same dentine specimens from each experimental group were used to decrease the variability between dentine specimens.

### Microbiological evaluation

#### Cultivation of *Streptococcus mutans* on dentine specimens

The bacterial strain used in this study was *Streptococcus mutans (S. mutans)* ATCC 700610. *S. mutans* were cultivated overnight in Brain-heart Infusion (BHI) broth (Sigma-Aldrich, Australia) at 37 °C and standardized to a concentration of 10^8^ CFU/mL. The dentine specimens were placed individually in an upright manner in sterile 24-well plates with 400 µL of BHI media. After that, 100 µL of the prepared bacterial suspension was added to each well and allowed to grow for 48 h under anaerobic conditions at 37 °C, 100 rpm for biofilm formation [40]. Following incubation, the dentine specimens with attached biofilms were subjected to the same HIFU exposure parameters (20W – 60s, 120s, and 180s). Further, to evaluate the anti-biofilm efficacy of HIFU against cariogenic biofilm, the following characterization methods were employed as described below.

#### MTT assay and colony forming unit (CFU)

The metabolic activity of *S. mutans* biofilms after HIFU exposure was evaluated using a 3-[4,5-dimethylthiazol-2-yl]-2,5 diphenyl tetrazolium bromide (MTT) assay kit (0.5 mg/mL MTT solution) (Sigma-Aldrich, Australia), following the manufacturer’s protocol. To release the bacteria remaining attached on the dentine surface, the treated specimens (*n = 3* per group) were ultrasonicated for 10 min in vials containing 500 µL of distilled water [34]. The resultant bacterial suspension was then pipetted in triplicate into a 96-well plate at a volume of 100 µL each. Then, 10 µL of MTT reagent was added to each well, followed by covering the plate and incubating it at 37 °C for 4 h. Following incubation, the reagent was aspirated, and 100 µL of the solubilizing solution was added to each well, followed by overnight incubation at 37 °C. Absorbance at 600 nm was measured using a spectrophotometer (SunriseTM, Tecan, Switzerland). To further confirm the viability of the bacteria post-treatment, a colony-forming unit (CFU) assay was conducted as previously described with minor adjustments [33, 34]. In brief, 100 µL of the ultrasonicated solution was serially diluted and plated on BHI agar plates, then incubated for 24 h at 37 °C anaerobically. The following equation was used to calculate the number of growing colonies [34]:

1 $$\begin{aligned}\text{CFU/mL } &=\text{ (Number of colonies }\cr&\quad\times\text{ Total dilution factor) }\cr&\quad \div\text{ Volume of culture plated in mL}\end{aligned}$$

#### Live & dead assay with confocal laser scanning microscopy (CLSM)

After exposure to HIFU, the dentine specimens (*n = 3 per group*) were stained for 15 min with LIVE/DEAD™ Baclight™ Bacterial Viability Kit (ThermoFisher Scientific, MA, USA) containing the dyes Propidium iodide (PI) and Syto9 following the manufacturer’s protocol. The samples were washed with 500 µL of PBS and fixed for 10 min using 4% paraformaldehyde. The specimens were visualized using Nikon A1RMP Multi-Photon Confocal Microscopy (Nikon Instruments Inc, NY, USA). The excitation wavelength for Syto9 and PI was centered at ~ 485 nm, whereas the fluorescence intensity was measured at ~ 530 nm (green) and ~ 630 nm (red), respectively. Three different locations on each specimen were scanned to get a representative image.

### SEM characterization of cariogenic biofilm

Dentine specimens from each group (*n = 3*) were subjected to a dehydration process involving a series of ascending ethanol concentrations and HMDS in a PELCO BioWave microwave operating at 250 W (Pelco BioWave, Ted Pella, Inc., USA). The dried specimens were affixed onto aluminium stubs using copper tape and platinum sputter coated (~ 3 mm) (Polaron SC7640, Quorum Technologies Ltd, U.K). Representative SEM images of each specimen were captured using Verios (XHR SEM, Thermo Fisher Scientific, USA) operated at an accelerating voltage of 10 kV.

#### Evaluation of cell proliferation

Dental pulp stem cells (hDPSC; Lonza BioSciences, USA) were used for the cell proliferation (*n = 3* per group/day) study to evaluate the effect of HIFU exposure on underlying DPSC cells [28]. The DPSCs were cultured in a dental pulp stem cell growth medium supplemented with human dental pulp stem cell growth supplement (DPSCGS), L-glutamine, Ascorbic acid, and Gentamicin/Amphotericin-B (GA) (Lonza BioSciences, USA). The culture was incubated with 5% CO_2_ at 37 °C under saturated humidity until 95% confluency was achieved. Cells between 3 and 5 passages were used. Upon confluency, the cells were seeded into 24 well plates at a seeding density of 0.06 × 10^6^ cells per well and incubated for 24 h before HIFU exposure. ACSD specimens (diameter < 4 mm) were positioned in a Transwell chamber (0.4 μm pore size; Corning) placed within the well plate, ensuring the cell growth medium contacted the underside of dentine discs. The upper surface of the dentine discs was exposed to varying HIFU parameters (Fig. 5a). After HIFU exposure, the metabolic activity of the cells was measured using acid phosphatase (APH) assay on days 0, 1, 3, and 7. For the APH assay, a complete buffer was prepared with 100 mM of sodium acetate, 1.1% Triton X-100, and 2 mg/mL of 4-nitrophenyl phosphate disodium salt hexahydrate (Cat. 71768, Sigma-Aldrich). After exposure to HIFU, the culture medium was removed, and the wells were washed with 500 µL of PBS (Cat. 20012043, ThermoFisher). Subsequently, 400 µL of complete APH buffer was added to each well and incubated for 1.5 h at 37 °C. The reactions were terminated by adding 40 µL of 1 N sodium hydroxide (NaOH) to each well, and then 100 µL of the solution was transferred to a 96-well plate in quadruplicate. Absorbance at 405 nm was promptly recorded within 10 min using a Sunrise absorbance reader (Cat. 16039400, Tecan, Grodig, Austria) [41].

For live and dead cell analysis (*n = 3*), after HIFU exposure, the cells were stained with NucBlue (Live) and Propidium iodide (Dead) for 30 min. After staining, the cells were washed with PBS (1x) and fixed with 4% paraformaldehyde for 10 min. Visualization was performed using a Nikon A1RMP Multi-Photon Confocal Microscope (Nikon Instruments Inc, NY, USA).

#### Dentine mineral density analysis

A micro-computed tomography (micro-CT) scanner (Nikon XT H225 ST CT, Nikon Corporation, Tokyo, Japan) was used to analyze the changes in mineral density (MD) in terms of Hounsfield units (HU). HU is a standard unit for X-ray CT density, where air and water are ascribed values of -1000 and 0, respectively, following a downward adjustment of 1000 units for conventional calibration. This method was utilized for HU calibration in Skyscan CTAn (Software version 1.14.4.1, Bruker micro-CT, Belgium), which was critical for ensuring the accuracy and consistency of HU measurements. Calibration based on air and water has demonstrated robustness over decades due to the linearity of the calibration curve across a range of X-ray densities. The calibration process provides a reliable reference, even for materials with densities higher than water. In addition, the thickness (µm) of the ACSD lesion removed upon HIFU exposure was determined. A window of 2 × 3 mm was outlined on each sound dentine (*n = 6* per group) (SD) as a treatment area. The dentine specimens were scanned using the same image settings for SD, ACSD, and after HIFU exposure. The image settings for micro-CT scanning were as follows: voltage: 90 kV, beam current: 75 µA, resolution: 8 μm, power: 6.8 W, exposure total: 1.42 s, gain: 24 dB, projection: 1800, without filters, and a helical CT scan was captured. To calculate the HU values, a water phantom of a similar size to the specimens was scanned with the same image settings. The mean grey values from the water volume of interest provide us with an HU calibration number, which is then applied to the study datasets. The resulting 2D projected images were reconstructed using Nikon’s CTPro 3D software (Version XT 6.7) with the following parameter settings: beam hardening correction: preset 2, volume attenuation scaling method: percentile. Average MD was estimated from the projection data using CTAn (Software version 1.14.4.1, Bruker micro-CT, Belgium), where regions of interest (ROIs) were outlined either in the entire lesion or different sections of the lesion. ROIs were also selected in the SD areas. For each region, twenty-five slices were selected to be stacked as a volume of interest (VOI), where the average MD in the Hounsfield unit (HU) was estimated. Average MD of SD, and demineralized lesions before and after HIFU exposure were obtained in each sample. Moreover, to evaluate the MD changes of demineralized lesions before and after HIFU exposure, the relative mineral density change (ΔMD) was calculated by:

2 $$\eqalign{& {\rm{\Delta MD}}\,{\rm{ = }}\, \cr & \,\,\,\,\left( {{\rm{M}}{{\rm{D}}_{after{\rm{ }}HIFU}}-{\rm{ M}}{{\rm{D}}_{Demin}}} \right)/{\rm{ M}}{{\rm{D}}_{SD}} \cr}$$

Where MD_after HIFU,_ MD_Demin,_ MD_SD_ are the average MD of VOIs within the demineralized dentine lesions after exposure to HIFU, ACSD demineralized state, and SD in each sample [42].

#### Characterization of dentine removal by HIFU

Confocal laser scanning microscopy (CLSM) was used to measure the variation in the surface reflectance an indication of surface mineral content, to detect the changes in the surface mineral intensity profiles of ACSD specimens (*n = 5* per group) before and after HIFU exposure. CLSM (Nikon A1 Si Confocal, Nikon Instruments Inc, NY, USA) microscope equipped with helium/neon-laser with a wavelength of λ = 488 nm, and a 4x dry lens objective was used to obtain a montage image of the dentine specimen surface in reflection mode, using the following parameters: stitching overlap: 5% and blending, Z series count: 5, maximum IP: 21.02 μm, range: 47.85 μm [43]. One-half of the dentine specimen surface with ACSD lesion was covered with aluminium foil, and the other half was subjected to varying HIFU exposures. The transition zone between the demineralized (ACSD) area and the HIFU exposed area was positioned in the centre of the field of view. The images were analyzed using the Nikon software (NIS Elements Version 3) and the surface intensity profile of the dentine specimens was recorded.

In addition, CLSM was used to determine the depth of dentine (ACSD) removal due to HIFU exposure. One-half of the dentine disc-shaped specimen surface with ACSD lesion was covered with aluminium foil, and the other half was subjected to varying HIFU exposures. After HIFU exposure, the dentine specimen surface (*n = 5* per group) was stained with 0.1% Rhodamine B solution (Sigma-Aldrich, Australia) for 24 h, and rinsed 3x with distilled water [44]. A laser (543 nm) was used to excite rhodamine B, with an emission spectrum captured between 555 and 655 nm (Nikon A1RMP Multi-Photon, Nikon Instruments Inc, NY, USA). Gain and offset were kept constant throughout all imaging with brightness adjustment made by fine-tuning the detector voltage. The vertical depth of dentine removal was quantified and analyzed using Nikon image analysis and ImageJ software (Fig. 7f).

#### Statistical analysis

Statistical analysis was performed utilizing SPSS Statistics (version 23.0, Armonk, USA). The data were represented as mean ± standard deviation and analyzed by one-way ANOVA followed by Tukey’s test for pair-wise comparison. For determining statistical significance, the threshold was set at *p* < 0.05. The required teeth sample size was calculated utilizing G*Power 3.1.9.7 (HHU, Düsseldorf, Germany) based on the specified parameters: for ANOVA with one-way analysis, using an effect size of f = 0.4, α err prob = 0.05, and power = 0.95. Consequently, the minimum sample size for the study was 125 samples.

## Results

### HIFU exposure alters the surface morphology of ACSD specimens

**Fig. 2:**
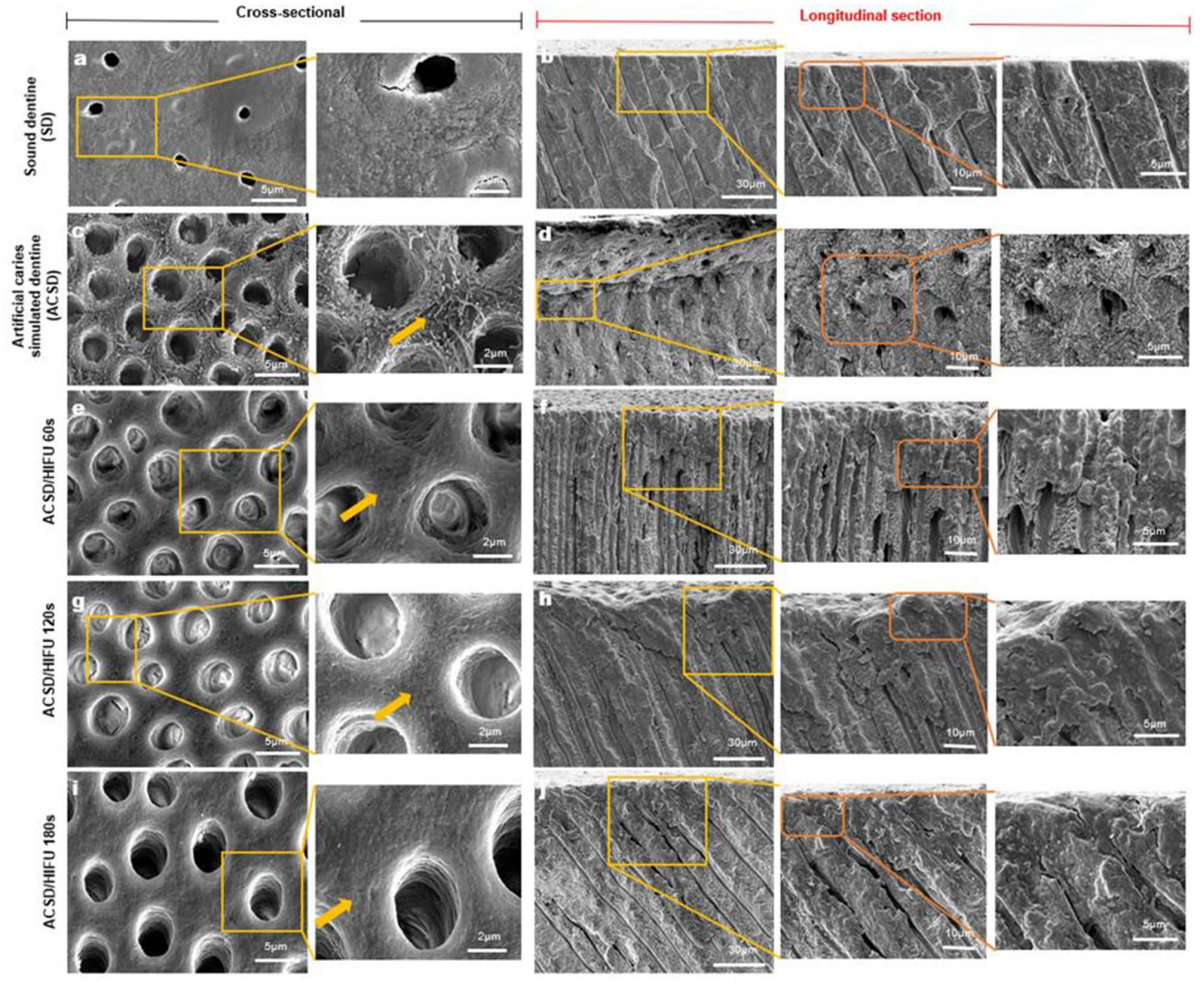
Scanning electron microscopy (SEM) images illustrating cross and longitudinal sections of sound dentine (SD) (**a**,** b**), artificial caries simulated dentine (ACSD) (**c**,** d**), and ACSD following HIFU exposure for 60s (**e**,** f**), 120s (**g**,** h**), and 180 s (**i**,** j**). The yellow arrow indicates progressive removal of demineralized collagen fibrils and residual smear layer with increasing HIFU exposure time (60s to 180s), culminating in a smoother intertubular dentine surface and wider dentinal tubules after HIFU exposure. Yellow insets highlight magnified views of the cross-sectional and longitudinal regions, showing detailed changes in the tubule structure after HIFU treatment, while orange insets in the longitudinal view emphasize structural changes such as the removal of the demineralized collagen fibril network and surface condensation post-HIFU exposure. Scale bar corresponds to the respective magnifications: 2500x − 30 μm; 5000x − 10 μm; 10,000x − 5 μm; 25,000x − 2 μm

The surface morphology of the ACSD specimens and ACSD/HIFU exposed specimens were distinguishable by SEM (Fig. 2c-i). After exposure to HIFU, the removal of the exposed demineralized collagen fibrils including any residual smear layer (Fig. 2c, d) was observed with increasing HIFU exposure time from 60s to 180s (Fig. 2e-j) respectively. Additionally, a smoother intertubular dentine surface texture having wider dentinal tubules is demonstrated following HIFU exposure for 180s (Fig. 2i, j). Both cross and longitudinal sections of HIFU exposed groups showed a condensed dentine surface free of the demineralized collagen fibrils network appearance (Fig. 2a, b).

### Enhanced mineral content and crystallinity of the ACSD specimen were observed following HIFU

**Fig. 3:**
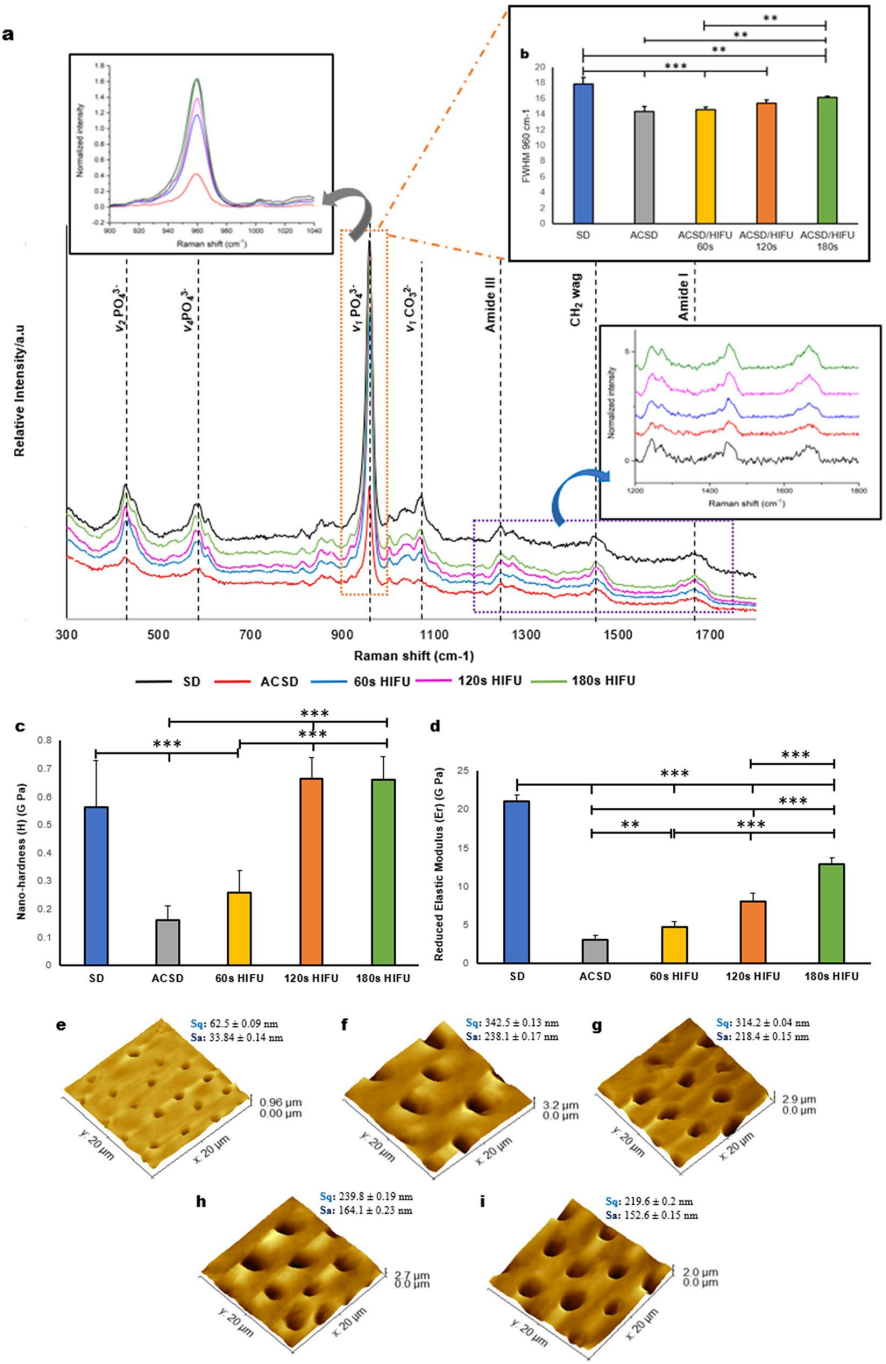
(**a**) Raman spectra (w/o background subtraction) of the sound dentine (SD) and experimental groups acquired in the region of 300–1800 cm^− 1^, showing the changes in the amide I, III, CH_2_ deformations, and PO_4_ groups after exposure to High-intensity focused ultrasound (HIFU). Normalized peak intensities at 960 cm^− 1^ and 1667 cm^− 1^ are shown in the intext box. Represents the mean ± standard deviation of the (**b**) Full-width-half maximum (FWHM) of phosphate peak width of the SD and experimental groups (*n* = 5) (*p < 0.05, **p < 0.01, ***p < 0.001). Bar graphs demonstrate the mean ± standard deviation of (**c**) the nano-hardness (H), and (**d**) the reduced elastic modulus (Er) of dentine specimens after HIFU exposure. (*p < 0.05, **p < 0.01, ***p < 0.001) (*n* = 5). Atomic force microscopy (AFM) images- 3D view (20 × 20) (**e**) Sound dentine (SD) (**f**) Artificial caries simulated dentine (ACSD) (**g**) ACSD/HIFU 60s (**h**) ACSD/HIFU 120s (**i**) ACSD/HIFU 180s. HIFU (from 60s to 180s) reduced the surface roughness of the ACSD substrate. Mean ± standard deviation of RMS roughness (Sq) and mean roughness (Sa) are depicted adjacent to each group (*n* = 5)

Representative Raman spectra obtained from the SD and experimental groups revealed significant differences in the intensities of characteristic peaks at 960 cm^− 1^, 1254 cm^− 1^, 1442 cm^− 1^, and 1667 cm^− 1^ (Fig. 3a). These peaks, particularly the phosphate peak at 960 cm^− 1^, which corresponds to the symmetric stretching mode of the phosphate group (v1 PO_4_^3−^) and serves as a key marker for mineral crystallinity, were more pronounced in HIFU-treated specimens compared to ACSD. The SD groups exhibited the highest intensity at 960 cm^− 1^, indicating greater mineral content and crystallinity, whereas the ACSD group demonstrated reduced peak intensity, reflecting diminished mineral content and lower crystallinity (Fig. 3b) [38, 39]. Exposure of the ACSD specimen to 180s of HIFU led to a significant increase in the intensity of the phosphate peak, suggesting partial restoration of mineral content in the treated specimens. However, despite this increase in intensity with 180s of HIFU, the FWHM analysis of the phosphate peak showed broadening, indicating that the crystallinity remained lower than that of SD (Fig. 3b).

### HIFU increased the nano-hardness and reduced elastic modulus of dentine specimens

Nano-indentation revealed a significant increase in the nano-hardness (H) values of 120s and 180s of ACSD/HIFU groups compared to ACSD and ACSD/HIFU 60s (*p* < 0.05) (Fig. 3c). Specifically, the ACSD specimens exposed to 120s and 180s of HIFU exhibited increased nano-hardness (H) compared to the ACSD group; however, this increase was not significantly different to SD. Furthermore, the reduced elastic modulus (Er) of all ACSD/HIFU specimens showed a significant increase compared to ACSD specimens (*p* < 0.05) in a time-dependent manner (Fig. 3d). The AFM images showed a smoother surface compared to the ACSD specimens and were dependent on HIFU exposure time, consistent with SEM images (Fig. 2), and this was further reflected on the RMS roughness (Sq) and mean roughness (Sa) values (Fig. 3e-i).

### HIFU demonstrated enhanced anti-biofilm activity and detachment of bacterial cells

**Fig. 4:**
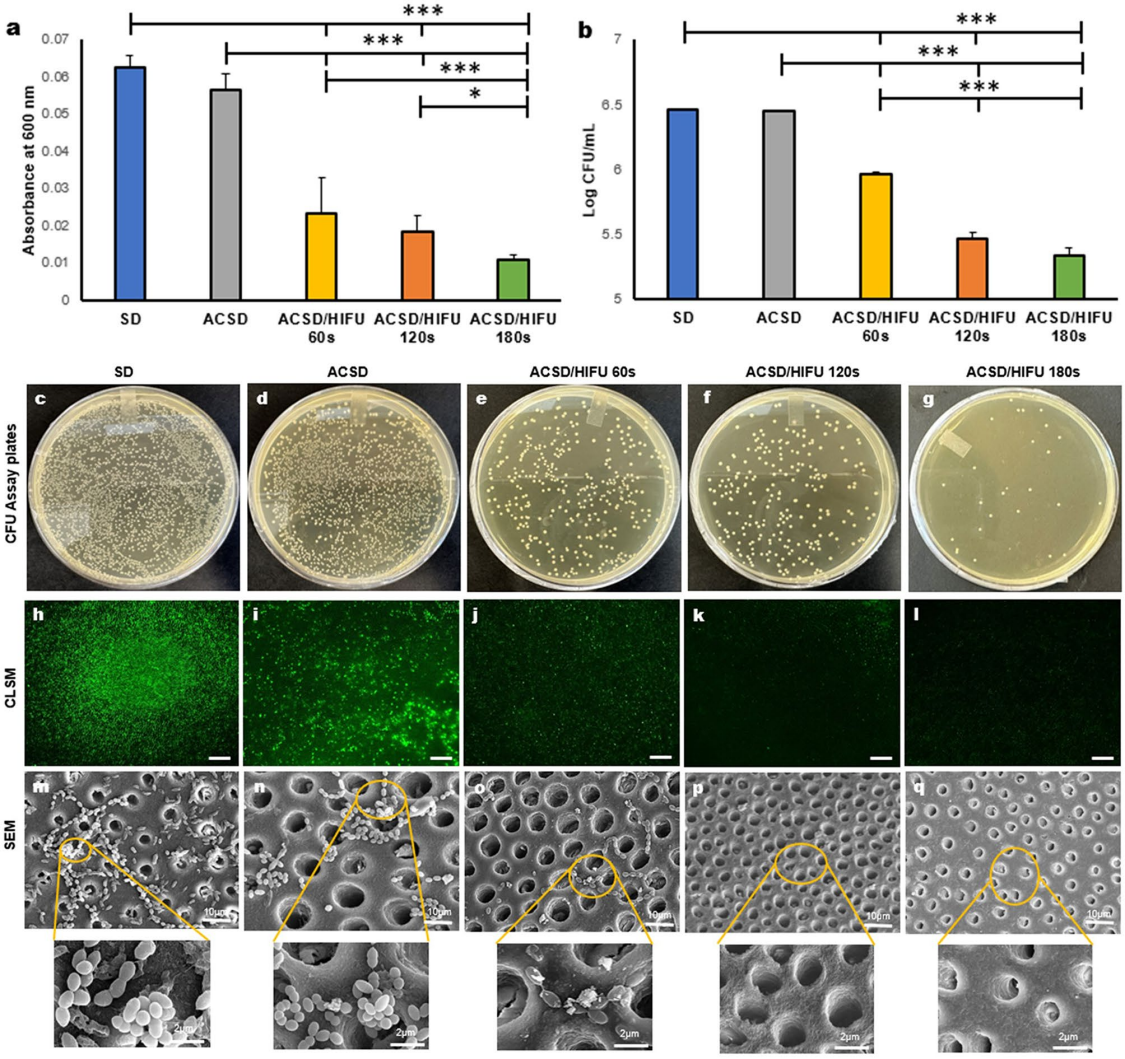
Bar charts of (**a**) 3-[4,5-dimethylthiazol-2-yl]-2,5 diphenyl tetrazolium bromide **(**MTT) assay measuring the % of bacterial viability among the experimental groups. (**b**) Log number of bacteria (colony forming units (CFUs)) in an mL after varying HIFU exposure. Data are presented as the mean ± standard deviation (*n* = 3) (*p < 0.05, **p < 0.01, ***p < 0.001). (**c-g**) CFU plates, which serve as visual representations of the antibacterial effectiveness exhibited by the experimental groups. (**h-l**) Representative confocal laser scanning microscopy (CLSM) images (10X mag) of the experimental groups after exposure to High-intensity focused ultrasound (HIFU); The presence of live bacteria (represented as green fluorescence) has decreased substantially upon HIFU exposure (All bars represent 100 μm). (**m-q**) Represent the scanning electron microscopy (SEM) images of the experimental groups. With increasing HIFU exposure, bacteria were completely removed/detached, and a condensed dentine structure was created similar to the results obtained when HIFU was exposed to the artificial caries simulated dentine (ACSD) surface without biofilm growth (Fig. 2e-j). Scale bar corresponds to the respective magnifications: 5000x − 10 μm; 25,000x − 2 μm

The metabolic activity of *S. mutans* biofilm for the experimental groups is depicted in Fig. 4a. Notably, there was a significant decrease in the relative metabolic activity observed in the ACSD/HIFU groups when compared to SD and ACSD (*p <* 0.05). In addition, the colony forming-unit (CFU) assay (expressed as log_10_ CFU/mL) (Fig. 4b) showed a significant reduction in CFU/mL when comparing the SD and ACSD to ACSD/HIFU groups (*p < 0.05*). Furthermore, Tukey’s test indicated that the log_10_ CFU/mL of remaining bacteria was significantly higher for the 60s ACSD/HIFU group when compared to the 120s and 180s of ACSD/HIFU groups (*p < 0.05*).

Representative confocal laser scanning microscope (CLSM) images show the presence of *S. mutans* biofilms on dentine specimens (Fig. 4h-l). The fluorescent image of SD (Fig. 4h), depicts all the bacteria emitting green fluorescence, indicating viability. Subjecting dentine specimens to demineralization (ACSD) resulted in fewer biofilm attachments on the surface (Fig. 4i). Exposure to HIFU from 60s to 180s (Fig. 4j-l) resulted in a demarcated decrease in biofilm attachment, with no biofilm observed in the ACSD/HIFU 180s group.

The SEM images of the SD revealed that *S. mutans* formed a dense biofilm structure firmly attached to the dentine specimen surface (Fig. 4m). Demineralized dentine (ACSD) specimens showed biofilm penetration into the dentinal tubules while remaining attached to the dentine surface (Fig. 4n). After 60s of HIFU exposure, the ACSD specimens displayed fewer biofilm attachments and compromised bacterial cell wall integrity (Fig. 4o). However, after 120s and 180s of HIFU exposure (Fig. 4p, q), no biofilm was attached to the dentine specimens. Additionally, exposure to 180s of HIFU created a textured surface on the dentine (Fig. 4q- magnified image), similar to those observed on dentine specimens without biofilm growth (Fig. 2i).

### HIFU promoted DPSC proliferation

**Fig. 5:**
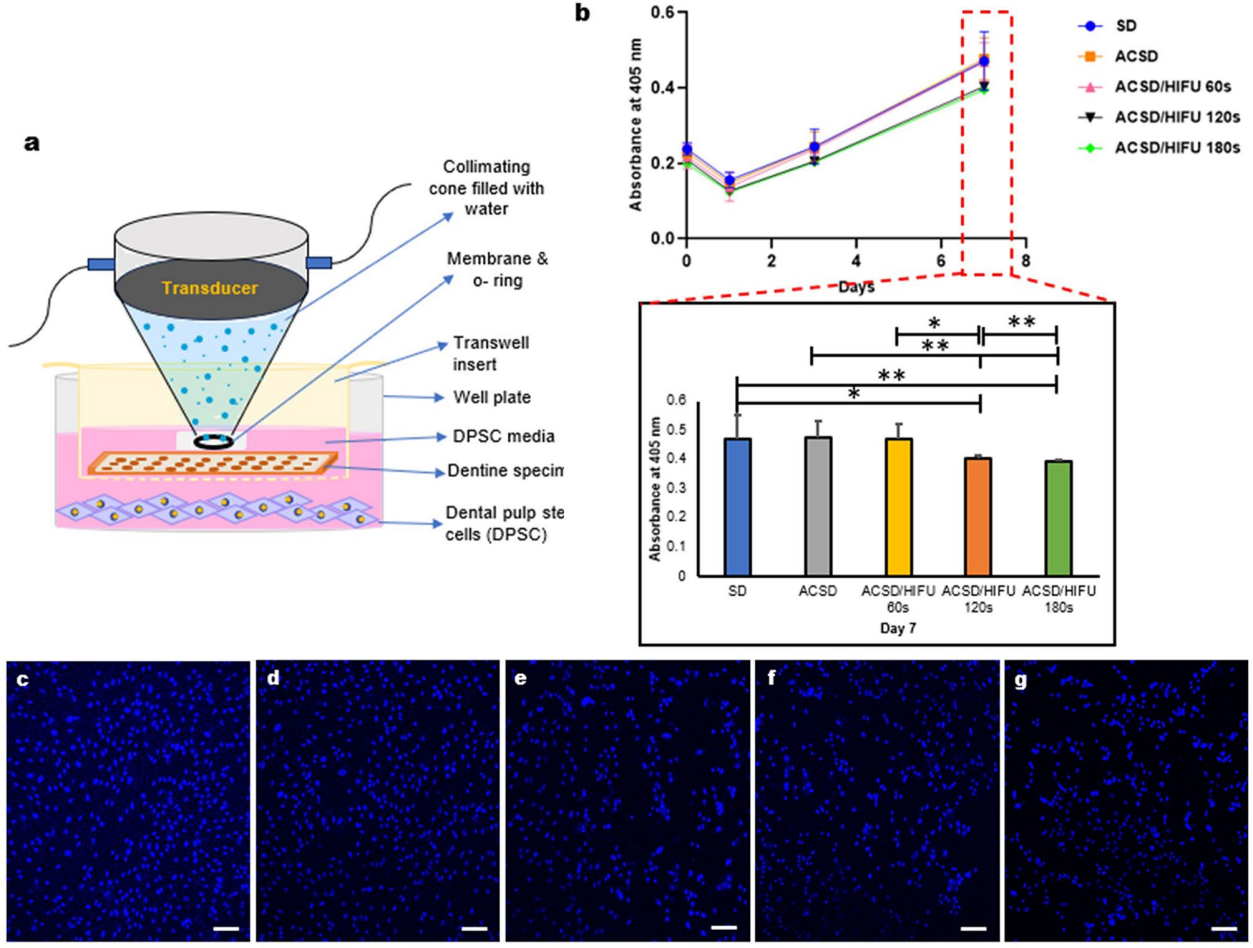
(**a**) Schematic diagram illustrating the experimental set-up used to investigate the effect of High-intensity focused ultrasound (HIFU) on the proliferation of underlying dental pulp stem cells (DPSCs). (**b**) Line chart representing the proliferative ability of DPSC (up to 7 days) after exposure to HIFU (*n* = 3). Intext bar chart representing proliferation of experimental groups on day 7 (*p < 0.05, **p < 0.01, ***p < 0.001). Confocal laser scanning microscopy (CLSM) images of DPSCs (**c**) sound dentine (SD) (**d**) artificial caries simulated dentine (ACSD) (**e**) ACSD/60s HIFU (**f**) ACSD/120s HIFU (**g**) ACSD/120s HIFU. Bar represents 100 μm

The proliferative/regenerative ability of the DPSC after HIFU exposure was evaluated over 0, 1, 3, and 7 days using APH assay (Fig. 5b). Despite an initial decrease in cell density on day 1, all groups exhibited a significant increase in cell density as the days progressed. Although cells exposed to 120s and 180s of HIFU showed slightly lower proliferation compared to the SD, ACSD, and ACSD/60s HIFU groups, the overall capacity of DPSCs to recover and sustain growth following HIFU exposure was preserved. Additionally, CLSM imaging was performed to visualize live and dead cells following HIFU exposure (Fig. 5c-g), revealing patterns consistent with those observed in the proliferation study.

### HIFU increased the mineral density of ACSD specimens

**Fig. 6:**
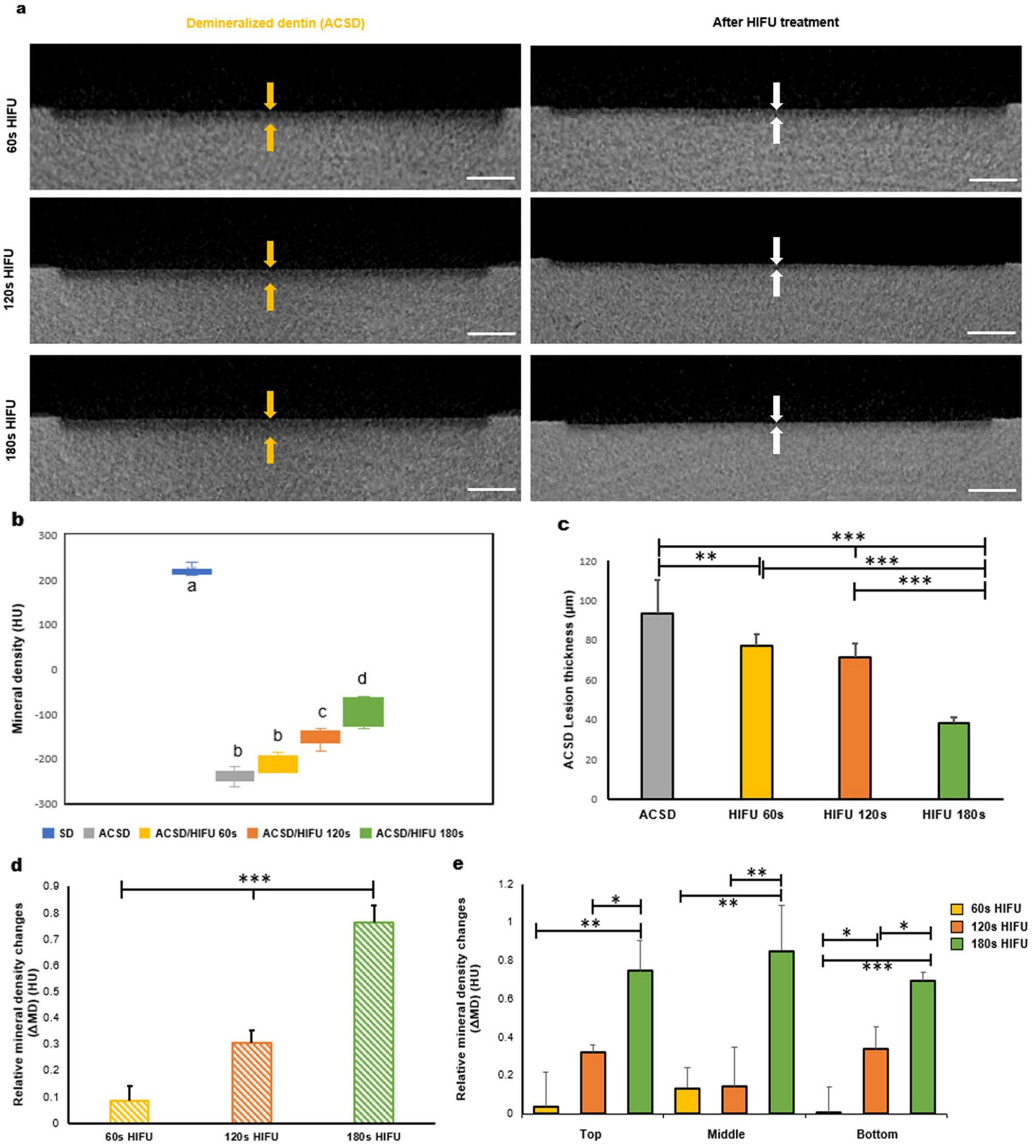
(**a**) Representative cross-sectional 2-D view of micro-computed tomography of artificial caries simulated dentine (ACSD) lesions before and after exposure to high-intensity focused ultrasound (HIFU). Arrows indicate the level of reduction in the thickness of the demineralized layer. Scale bars represent 300 μm. (**b**) Boxplots comparing the mineral density of the sound dentine (SD), artificial caries simulated dentine (ACSD), 60s HIFU, 120s HIFU, and 180s HIFU. (**c**) Represents the lesion thickness (µm) of the experimental groups. (**d**) Comparison of the relative mineral density change (ΔMD) of the entire ACSD after exposure to varying HIFU parameters. (**e**) Bar graph comparing the ΔMD in the top, middle, and bottom of the ACSD sample after exposure to HIFU parameters. (*p < 0.05, **p < 0.01, ***p < 0.001) (*n* = 6)

Micro-CT cross-sectional slices demonstrate a substantial reduction in demineralized (ACSD) lesion density with increased HIFU exposure from 60s to 180s (Fig. 6a). A significant reduction in the mineral density (MD) was observed in ACSD (~ -237.184 ± 16.41 HU) when compared to SD (~ 224.27 ± 19.43 HU) (Fig. 6b). However, exposure to 120s (~ -152.77 ± 20.35 HU) and 180s (~ -94.95 ± 41.01 HU) of HIFU led to a significant increase in the MD compared to ACSD (*p* < 0.05) (Fig. 6b). In addition, the variation in ACSD lesion thickness (µm) removed upon HIFU exposure was measured and represented in Fig. 6c. A significant reduction in the lesion thickness can be seen when ACSD specimens (~ 93.64 ± 16.83 μm) were exposed to 180s HIFU (38.59 ± 1.32 μm) (*p* < 0.05). ACSD specimens exposed to 180s of HIFU showed an increase in the relative MD change when compared to 60s and 120s of HIFU exposure (*p* < 0.05) (Fig. 6d). This increase in the relative mineral density change was not limited to the top one-third of the dentine specimen containing the ACSD lesion but was also observed in the middle and bottom one-third of the dentine specimens containing the ACSD lesion (*p* < 0.05).

### HIFU demonstrated dentine removal effect

**Fig. 7:**
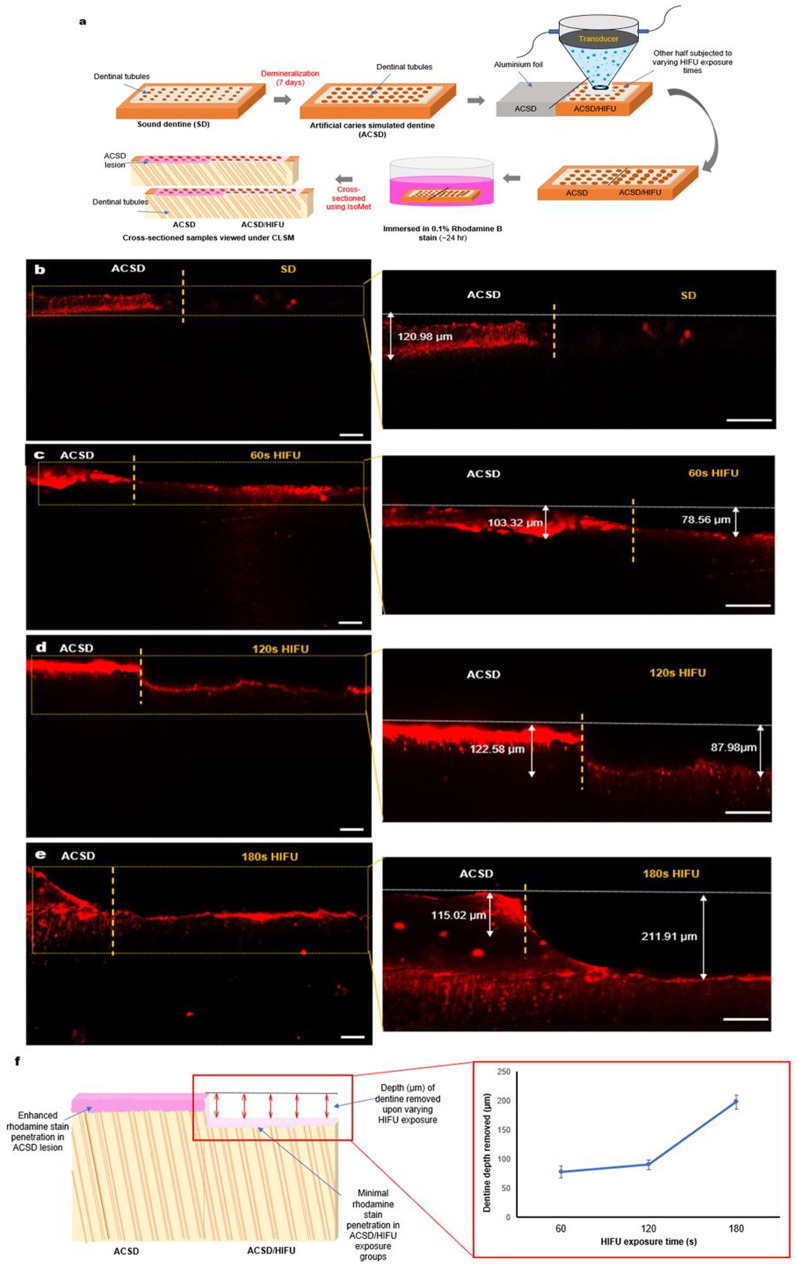
(**a**) Schematic diagram demonstrating the procedure for evaluating the depth of dentine removed upon high-intensity focused ultrasound (HIFU) exposure. (**b-e**) Representative confocal laser scanning microscopy (CLSM) images of dentine specimens stained with rhodamine B after exposure to varying HIFU parameters to measure the vertical height (depth) of dentine removal. The white double-line arrow represents the depth of the dentine removed (µm). All scale bars represent 100 μm. Magnified image scale bars represent 200 μm. (**f**) Schematic illustration depicting measurement of dentine depth removed (µm). Line graph representing HIFU exposure time (s) vs. dentine depth removed (µm) (*n* = 5)

Figure 7a represents the schematic diagram for evaluating the depth of dentine removed upon HIFU exposure using CLSM. The ACSD demineralized lesions were easily distinguishable from the SD and ACSD/HIFU-exposed groups due to the enhanced penetration of rhodamine B into the pore space produced as a result of excessive mineral loss and exposure of interfibrillar spaces (Fig. 7b-d). The penetration of rhodamine B stain into the ACSD lesion can be seen up to ~ 116.44 ± 9.06 μm (Fig. 7b-d). Increasing the HIFU exposure from 60s to 180s, revealed a significant increase in the depth of dentine removed from ~ 77.96 ± 10.01 μm to ~ 197. 3398 ± 12.52 μm (Fig. 7c-e; f).

**Fig. 8:**
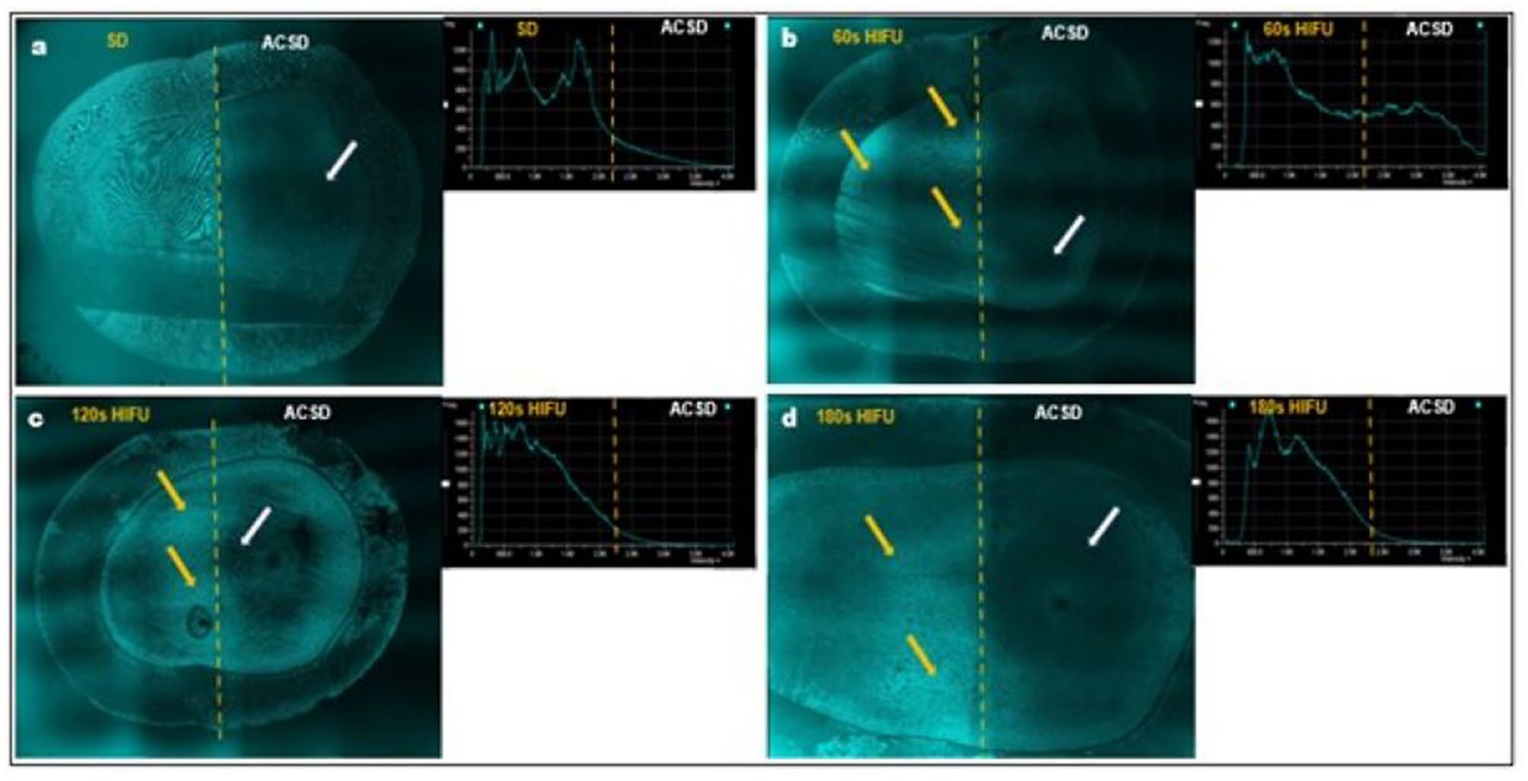
(**a-d**) Representative confocal laser scanning microscopy (CLSM) montages illustrate the variation in the surface reflectance intensity profile of artificial caries simulated dentine (ACSD) specimens to varying high-intensity focused ultrasound (HIFU) exposures. (Yellow arrows indicate the increase in surface reflectance intensity with increasing HIFU exposure, while white arrows indicate lower surface density/reflectance observed in demineralized (ACSD) areas). All CLSM images were captured at 4x magnification (*n* = 5)

In addition, CLSM operated in reflection mode was used to gain montage images of the ACSD/HIFU-exposed specimens (Fig. 8a-d). The ACSD specimen surface profile appeared as a dark area, which seems to be opaque, whereas the SD and specimens exposed to increasing HIFU exposures demonstrated an increased surface reflectance intensity attributing to an increase in the mineral content. HIFU exposure for 60s slightly increased the surface intensity profile compared to ACSD (Fig. 8b). However, exposure to 120s and 180s resulted in a well-demarcated increased surface intensity profile with distinguished peaks when compared to ACSD (Fig. 8c, d).

## Discussion

High-intensity focused ultrasound (HIFU) was previously introduced as a minimally invasive technique for dentine surface conditioning to optimize resin-dentine bonding without resorting to acid-etching [27]. This approach preserves collagen integrity and demonstrates a significant time-dependent ablation effect [28]. Further investigations have reported HIFU’s non-deleterious impact on the organic and collagen structure of dentine using multi-scale characterization methods [28]. In addition, owing to its unique characteristics of focused intense acoustic waves, HIFU was employed as an intervention tool to treat several medical conditions through various mechanisms of action such as tissue destruction, thermal/non-thermal ablation [45], drug-delivery [31], sonoporation, stone fragmentation, and immunomodulation [30, 45, 46]. Therefore, this study embarks on exploring the potential of HIFU in dentistry as a minimally invasive tool for microscale manipulation and excavation of carious dentine lesions. Having a precise controllable tool capable of selectively removing carious dentine substrates at the microscale, while exhibiting anti-biofilm properties, holds substantial clinical significance in restorative and preventive dentistry.

Instead of employing phosphoric acid demineralization to create a demineralized dentine substrate [28, 47, 48], this study adopted a specified protocol utilizing a demineralizing solution composed of acetic acid, CaCl_2_, and KH_2_PO_4_ over a period of 7 days to induce artificial demineralized lesion simulating caries-affected dentine (ACSD) as study substrate model [37]. Concurrently, while investigating the interaction of HIFU with the ACSD model, the associated thermal changes were characterized to ensure that the temperature rise within the pulp space remained within acceptable physiological biological tolerance (Fig. 1b II).

Utilizing a comprehensive array of characterization techniques including SEM, Raman spectroscopy, AFM, and nano-indentation, this study investigates the potential of HIFU in selectively removing soft dentine collagen. A discernible time-dependent effect was observed on ACSD specimens upon HIFU exposure. Extending HIFU exposure from 60s to 180s resulted in the removal of demineralized dentine collagen, producing a smoother intertubular dentine surface with widened dentinal tubules (Fig. 2). Raman spectroscopy (Fig. 3a, b) further elucidated the chemical composition of dentine specimens, revealing augmented peak intensities in phosphate bands and amide bands in HIFU-exposed groups compared to ACSD specimens. Despite these enhancements, the crystallinity, as indicated by the FWHM of the phosphate peak, did not fully revert to that of sound dentine, indicating that while HIFU at 180s partially restores mineral content and the organic matrix, it does not fully restore the mineral to its original crystalline structure. These spectroscopic findings were complemented by increased nano-hardness (Fig. 3c), reduced elastic modulus (Fig. 3d), and decreased surface roughness (Fig. 3e-i) values. The dual effect of enhancing mineral deposition while maintaining a more amorphous phase could have implications for the mechanical properties of the treated tissue, potentially offering benefits in terms of toughness and resilience, though not fully mimicking the properties of sound dentine. Moreover, changes in mineral density (ΔMD) were observed with prolonged HIFU exposure, resulting in a significant reduction in the demineralized layer and an increase in mineral density at 120s and 180s (Fig. 6). The mechanism(s) by which HIFU removes soft dentine could be likely attributed to both thermal and/or mechanical effects [45, 49]. The localized application of acoustic waves generates heat, causing tissue ablation and necrosis, while the mechanical effects induce cavitation, leading to the formation and collapse of microbubbles within the tissues. These rapid pressure changes create shear forces that disrupt tissue’s structure, facilitating their removal [45, 49]. Additionally, acoustic streaming generated by HIFU contributes to the physical displacement of tissues, further aiding the ablation process [45, 46]. The HIFU/tissue interaction depends on the properties of the underlying tissue and the specific parameters of HIFU exposure [50]. Therefore, fine-tuning this relationship could lead to more controlled and selective tissue ablation. This pioneering investigation confirms that HIFU can effectively remove carious-simulated dentine (ACSD) in a time-dependent manner, though the removal effect diminishes with depth. The observed decrease in removal efficiency with depth may be attributed to wave attenuation, tissue absorption, and increasing mineral content with the associated changes in tissue properties including elastic modulus.

Therefore, as a step towards future translation of this approach into clinical application, it is imperative to explore any correlation between HIFU exposure parameters and the depth of dentine removal. In this study, we examined the relationship between HIFU exposure time and removal depth, while standardizing other HIFU exposure parameters. The above results indicated that increasing the HIFU exposure time leads to increased dentine depth removal. To further explore this phenomenon, we utilized CLSM as a preliminary tool to measure the depth of HIFU-induced dentine removal, using rhodamine B stain (Fig. 7b-e). As the duration of HIFU exposure increased, there was a substantial increase in the depth of dentine removed, reaching up to ~ 197. 3398 ± 12.52 μm with 180s of HIFU exposure (Fig. 7e). Following HIFU exposure, the CLSM images showed a thinner residual rhodamine infiltrated- layer, indicating the removal of primarily ACSD dentine and leaving a mineral-rich layer behind (Fig. 7d, e). These observations confirm that the HIFU excavation process is time-dependent and significantly influenced by the tissue’s characteristics and HIFU exposure parameters. Additionally, reflective mode CLSM images further supported these findings, showing increased surface reflectance intensity in ACSD/HIFU exposed specimens, indicative of enhanced mineral content, particularly following 180s of HIFU exposure (Fig. 8). While our characterization was limited to a two-dimensional analysis, future studies incorporating three-dimensional analyses are warranted to provide a more comprehensive understanding of the nature of tissue removal. Within this study, it was discerned that exposure to HIFU for up to 180s, within the specified parameters, resulted in effective microscale removal of carious-simulated dentine.

For the successful translation of a new technology into dental practice, it is imperative to consider the biological implications. Evaluating the biological effects of HIFU on underlying DPSCs is crucial for its potential application in microscale excavation or removal of deep carious dentine, aiming to minimize potential adverse effects on pulp tissues. Numerous studies have demonstrated that ultrasound stimulation enhances protein and collagen synthesis [51] and increases nodule formation [52, 53] at lower exposure times. In this study, we assessed the proliferative capacity of DPSCs at intervals of 0, 1, 3, and 7 days post-HIFU exposure, and observed a notable increase in cell density over time (Fig. 5b). Additionally, CLSM visualization of hDPSC revealed intact cellular morphology (Fig. 5c-g). These findings suggest that HIFU treatment does not impair the proliferative potential of hDPSC. However, further investigations are necessary to verify these observations and to elucidate the mechanisms by which HIFU facilitates DPSC proliferation. It is worth noting that the associated temperature rises within the pulp space following HIFU exposure remained within the physiological tolerance of pulp tissues (Fig. 1b II).

The microbiological impact is of paramount importance in addressing dentine carious lesions, typically associated with microbial colonization. Recognizing this, in our investigation, we evaluated the bactericidal effectiveness of HIFU by measuring two key parameters: metabolic activity, quantified via MTT assay, and colony-forming ability (CFU). Increasing HIFU exposure, particularly at 180s, demonstrated enhanced efficacy in biofilm removal. This effectiveness was confirmed by a reduction in the metabolic activity (Fig. 4a) and decreased capacity for bacterial colony formation (Fig. 4b). The observed bactericidal effect following HIFU exposure is likely attributable to the generation of mechanical shock waves, which disrupt bacterial cell walls and induce the production of reactive oxygen species (ROS) such as OH·, HO_2·,_ O·, and H_2_O_2_ [35, 54–56]. Additionally, these shock waves increase the conversion of acoustic energy to heat, facilitating its dissemination throughout the sample *via* thermal diffusion [35, 49, 54, 57]. Furthermore, we utilized CLSM (Fig. 4h-l) to examine the presence of live bacteria on the dentine substrate, and SEM (Fig. 4m-q) was employed to investigate the biofilm structure and bacterial adherence to the dentine substrate. Treatments involving HIFU with prolonged exposure times demonstrated an augmented detachment of biofilm, possibly due to the disruptive capacity of acoustic waves on biofilm structure (28, 55, 56), thus dislodging bacteria from the surface and potentially resulting in the absence of red bacterial cells in CLSM images. Furthermore, owing to bacteria’s limited adaptability to sustained mechanical waves, HIFU treatment offers a reduced risk of resistance development [58, 59]. This highlights HIFU’s potential as an effective drug-free antimicrobial strategy in the treatment of dentine carious lesions.

These findings collectively highlight the potential of HIFU for the micro-level removal and excavation of carious-simulated dentine (ACSD) lesions, while demonstrating effective anti-biofilm properties. If further developed and validated this minimally invasive approach offers a targeted strategy for managing carious lesions at a microscale level. Our investigation focused on correlating the depth of dentine removal with HIFU exposure time. Future studies should refine this approach by validating additional HIFU parameters, such as intensity, frequency, and focal point/depth profile, using a customized dentine substrate with varying degrees and depths of demineralization to simulate a wider spectrum of demineralized dentine lesions. This will help develop a fully controlled tool for the selective removal of soft dentine. The non-ionizing characteristic of HIFU minimizes the risk of damage to adjacent tissues and permits repeated treatments [59] due to its ability to achieve controlled, selective, and localized combined tissue ablation and alteration effects.

## Conclusion

This study introduced the potential of high-intensity focused ultrasound (HIFU) as a minimally invasive tool for microscale manipulation/excavation of artificial carious-simulated dentine (ACSD) substrates. Furthermore, HIFU exposure at 250 kHz and 20 W in continuous mode for 180 s effectively removed the ACSD substrate while demonstrating significant anti-biofilm efficacy, which was positively correlated with exposure time.

## Data Availability

All raw data will be made available by the corresponding author upon reasonable request.
